# Symptom burden profiles and applied medication strategies in the days before and after initiation of palliative sedation in advanced cancer patients: an international prospective multicentre observational study (PALSED)

**DOI:** 10.1007/s00520-026-11358-7

**Published:** 2026-10-27

**Authors:** Maaike Rijpstra, Evelien Kuip, Alazne Belar, Séverine Marie Surges, Claudio Adile, Rocío Rojí, Sebastiano Mercadante, Lukas Radbruch, Johan Menten, Carlos Centeno, Jeroen Hasselaar, Kris Vissers

**Affiliations:** 1 Department of Anaesthesiology, Pain and Palliative Medicine, Radboud University Medical Centre, Geert Grooteplein 10, Nijmegen, 6500 HB The Netherlands; 2 Department of Primary and Community Care, Radboud University Medical Centre, Nijmegen, The Netherlands; 3 Department of Medical Oncology, Radboud University Medical Centre, Nijmegen, The Netherlands; 4 ATLANTES Global Observatory of Palliative Care, Institute for Culture and Society, University of Navarra, Pamplona, Spain; 5 IdISNA‑Instituto de Investigación Sanitaria de Navarra, Palliative Medicine, Pamplona, Spain; 6 Department of Palliative Medicine, University Hospital Bonn, Bonn, Germany; 7 Main Regional Centre of Supportive/Palliative Care, La Maddalena Cancer Centre, Palermo, Italy; 8 Department of Oncology, Laboratory of Experimental Radiotherapy, University Hospital and Catholic University, Louvain, Belgium; 9 Symptom Control and Palliative Medicine, Clinica Universidad de Navarra, Pamplona, Spain

**Keywords:** Palliative sedation, Advanced cancer, Symptom assessment, Medication strategies, End-of-life care, Prospective studies

## Abstract

**Background:**

Advanced cancer patients may experience refractory suffering at the end of life despite optimal symptom management. When symptoms can no longer be controlled, or not within an acceptable timeframe, palliative sedation may be indicated. However, evidence on symptom severity and pharmacological strategies before sedation remains limited. This study aimed to characterize symptom burden and medication use in the 24 h before and after initiation of palliative sedation.

**Methods:**

In this prospective observational study, data were collected from adult cancer patients receiving palliative sedation at seven European care settings (hospital wards, hospices, and PCUs). Healthcare professionals documented symptoms, pharmacological management, and sedation characteristics. Symptom prevalence and severity was assessed by patients or proxies using the Edmonton Symptom Assessment System (ESAS).

**Results:**

Among 80 patients with palliative sedation, multiple refractory symptoms were common at initiation; 81% had two or more symptoms. Dyspnoea (23%), delirium (18%), and exhaustion (16%) were the primary symptoms prompting sedation. ESAS data (*n = *66) showed high prevalence of drowsiness, appetite loss, tiredness, and reduced wellbeing (73–91%), with severe burden scores (≥ 7) in 63–78%. Prior palliative sedation, patients with fewer symptoms (1–2) received a wider range of medications, including corticosteroids, cardiovascular agents, and anti-infectives, while those with ≥ 3 symptoms more often received anxiolytics, hypnotics, and sedatives. After initiation, most medications were discontinued; opioids were continued, and midazolam was the predominant continuously administered sedative.

**Conclusion:**

Refractory suffering often reflected overall clinical decline rather than isolated symptoms. While pre-sedation medication use varied, post-initiation strategies were consistent across patients.

**Supplementary Information:**

The online version contains supplementary material available at https://doi.org/10.1007/s00520-026-11358-7.

## Background

Patients with advanced cancer commonly experience multiple distressing symptoms near the end of life, including fatigue, pain, and loss of appetite [1]. These symptoms typically worsen in the final months [2] or weeks [1, 3]. Despite standard management strategies, symptoms may become difficult to control or refractory [4], especially when physical, psychological, or existential distress coexist [5].

Refractory burden may result from limited treatment options, reduced drug efficacy, or delayed response. Near death, symptoms such as breathlessness, delirium, and existential suffering may become refractory, with reported prevalences of 57–83% [6]. When such symptoms lead to refractory burden and death is imminent, palliative sedation may be indicated [7].

Palliative sedation aims to relieve intolerable suffering by proportionally reducing consciousness [8]. Delirium, dyspnoea, and pain are the most frequent reasons for initiating palliative sedation [7], though decisions often reflect a combination of symptoms [7, 9, 10]. However, data on symptom severity and clustering at initiation and medication strategies for complex symptom burden remain limited.

This study describes refractory symptom burden leading to palliative sedation in advanced cancer patients, and examines evolution of pharmacological management before and within 24 h after initiation of sedation. Also, treatment patterns were compared between groups of patients with fewer versus more symptoms.

## Methods

This study is part of the prospective multicentre observational study from European H2020 project Palliative Sedation, described in detail elsewhere [11]. In brief, adults with advanced cancer admitted to seven European palliative care settings (hospital wards, hospices, and palliative care units) between April 2021 and April 2023 were invited to participate. Eligible patients had a limited life expectancy and (possible) development of refractory suffering, indicating a potential need for palliative sedation as assessed by the treating healthcare team. After informed consent, baseline assessments were performed, and Participants were prospectively followed during admission until discharge or death, with additional observations of the sedation process if decision-making regarding palliative sedation began. Further details on the study design, eligibility criteria, and study procedures are reported elsewhere [11, 12]. Study registration: ClinicalTrials.gov, NCT04719702 (registered January 22, 2021).

For the present analysis, initiation of palliative sedation was defined as the actual start of sedation, with explicit confirmation by the treating healthcare professional that the intervention was intended as palliative sedation. Patients meeting this definition were included in the analytical sample. Symptom burden and medication profiles were obtained through standardized data collection tools across all care settings, ensuring consistency and minimizing measurement bias.

### Outcome measurements

#### Refractory symptom(s) reported by healthcare professionals

Healthcare professionals recorded the refractory symptom(s) prompting initiation of sedation selecting symptoms from a predefined checklist. All symptoms present at initiation were registered and ranked by perceived importance. The highest-ranked symptom was considered the primary indication. Pairwise symptom combinations were analysed to identify frequent co-occurrences. For ‘exhaustion’ and ‘existential distress’, healthcare professionals could provide free-text descriptions to capture physical, psychological, and spiritual dimensions. ‘Exhaustion’ was used as an umbrella concept referring to profound physical and mental fatigue. The free text allowed healthcare professionals to describe their observations of both concepts in terms commonly used in their clinical practice.

#### Symptom burden assessed by patients or proxies

Patient symptom burden was assessed using the Edmonton Symptom Assessment System (ESAS; nine items, 0–10 numeric rating scale) [13]. To reflect routine clinical practice within each participating site, we used the locally implemented validated ESAS versions. In the Dutch validation of the ESAS (Utrecht Symptom Diary, USD), the drowsiness item was omitted [14]. This item was therefore missing in assessments of those patients.

Symptoms were categorized as absent (0), present (≥ 1), and mild (1–3), moderate (4–6), or severe (≥ 7) [13]. ESAS was completed at baseline and repeated if more than two days had elapsed before the decision for palliative sedation. When patients were unable to self-report, proxy ratings from a familiar healthcare professional were used. To evaluate potential effects of reporting source, we conducted a sensitivity analysis restricted to patient-completed assessments.

#### Medication use 24 h before and after palliative sedation initiation

Medication registration focused on drugs administered within the 24 h before and after initiation of palliative sedation. Medications were categorized according to the Anatomical Therapeutic Chemical system (ATC/DDD Index 2025, WHO [15]). Under the category cardiovascular agents, we included antihypertensives, diuretics, beta-blockers, calcium-channel blockers, and renin–angiotensin system agents. Anxiolytics, hypnotics, and sedatives administered prior to palliative sedation were primarily prescribed for symptom control (e.g., anxiety or sleep disturbance), whereas during and after initiation they were explicitly used for sedation and therefore reported as part of the palliative sedation strategy.

#### Palliative sedation characteristics and strategies

Palliative sedation was monitored from initiation until death. Recorded variables included: time from admission to sedation initiation, achievement of adequate sedation, and duration. Time to adequate sedation was defined as the time from initiation of palliative sedation until the treating healthcare professional considered the level of sedation to be adequate according to regular clinical practice. Strategy variables included modality of palliative sedation at start (intermittent or continuous), sedative agents, and hourly midazolam dose during the first six hours.

### Hypotheses and objectives

We hypothesized that clinician-observed symptom profiles reflect the overall level of refractory burden and, consequently, influence treatment patterns, including medication diversity, treatment discontinuation, and the applied palliative sedation strategies. The objectives of this study were to:

(1) describe the symptom burden at the time of indication for sedation from both patient and healthcare professionals perspectives.

(2) assess whether medication use before and after the initiation of palliative sedation differed between groups with fewer versus more refractory symptoms; and.

(3) explore whether the number of symptoms present was associated with variations in the characteristics of sedation.

### Statistical analysis

Analyses were performed using SPSS v29 and RStudio v2024.04.2.Categorical variables were summarized as counts and percentages; continuous variables as mean (SD) or median [IQR]. Medication profiles and sedation characteristics were analysed overall and compared between groups of patients with fewer (1–2, FSG) versus more symptoms (≥ 3, MSG). Group differences were tested using Mann–Whitney U for continuous variables and chi-square or Fisher’s exact tests for categorical variables. Primary outcomes included medication class, and for palliative sedation: time variables, modality, sedatives, and initial dosages.

## Results

A total of 248 patients were enrolled, of whom nine were followed during two separate admissions, resulting in 257 admissions. Follow-up ended in 115 admissions due to discharge to another care setting. Among the remaining 142 admissions, 62 patients died without initiation of palliative sedation whereas this was initiated in 80 patients before death.

Of the 80 patients in which palliative sedation was initiated, 46% were male and the mean age was 69 years. The most common primary diagnoses were malignancies of the digestive (35%), respiratory (15%), and female or male genital organs (14%). Patients were admitted to hospices (46%), oncology wards (30%), or PCUs (24%). For all baseline data see Table 1.

**Table 1:** Baseline characteristics total study sample (*n = *80)

Setting	
*Hospice*	37 (46%)
*Hospital ward*	24 (30%)
*Palliative Care Unit*	19 (24%)
Gender, *male*	37 (46%)
Age, *mean (SD)*	69 (12.2)
Malignant neoplasm of	
*Digestive organs*	28 (35%)
*Respiratory organs*	12 (15%)
*Breast*	10 (13%)
*Female-or male genital organs*	11 (14%)
*Other*	19 (24%)
Amount of comorbidities	
*0*	20 (25%)
*1*	23 (29%)
*2*	25 (31%)
*3 or more*	12 (15%)
ECOG Performance Status^a^	
*2 or lower*	8 (10%)
*3*	32 (40%)
*4*	38 (48%)
*Missing*	2 (3%)

*SD* standard deviation, *ECOG* Eastern Cooperative Oncology Group (Performance Status)

a. ECOG PS categorical: grades 0 (fully active) to 4 (completely disabled)

### Refractory symptoms prompting palliative sedation

**Fig. 1:**
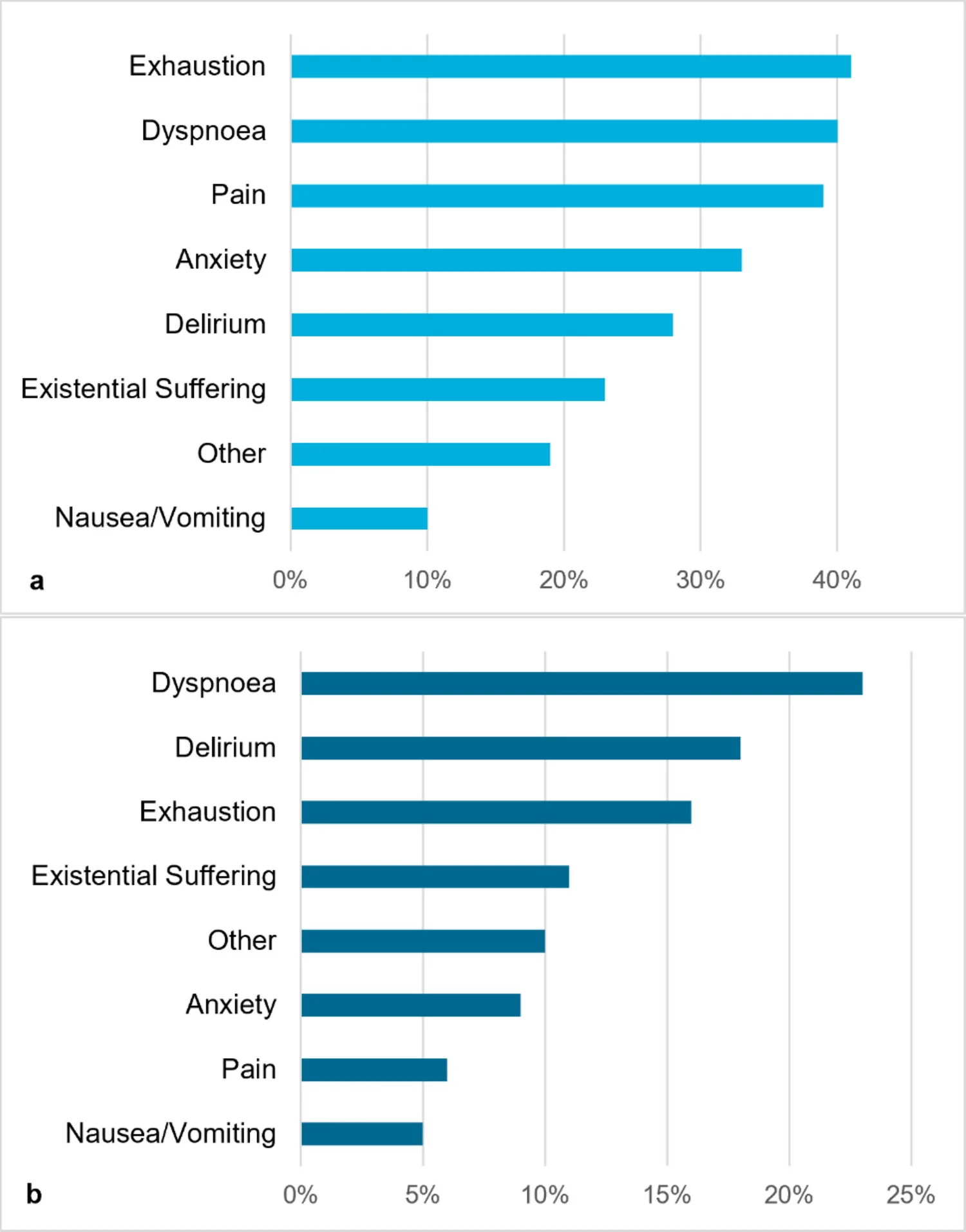
HCP reported refractory symptoms (occurrence and highest ranked) at start of PS. **a** All reported refractory symptom(s) present at start of PS in total sample (*n = *80); **b** Highest ranked refractory symptom leading to initiation of PS, order of present symptoms not ranked in 2 patients (3%); *PS* palliative sedation, *HCP* healthcare professional

In Fig. 1a-b the occurrence of refractory symptom(s), and the highest ranked symptom by healthcare professionals are summarized. According to healthcare professionals, exhaustion (41%), dyspnoea (40%), and pain (39%) occurred most frequently, while dyspnoea (23%), delirium (18%), and exhaustion (16%) were most often ranked as the primary reason for initiation of palliative sedation.

**Fig. 2:**
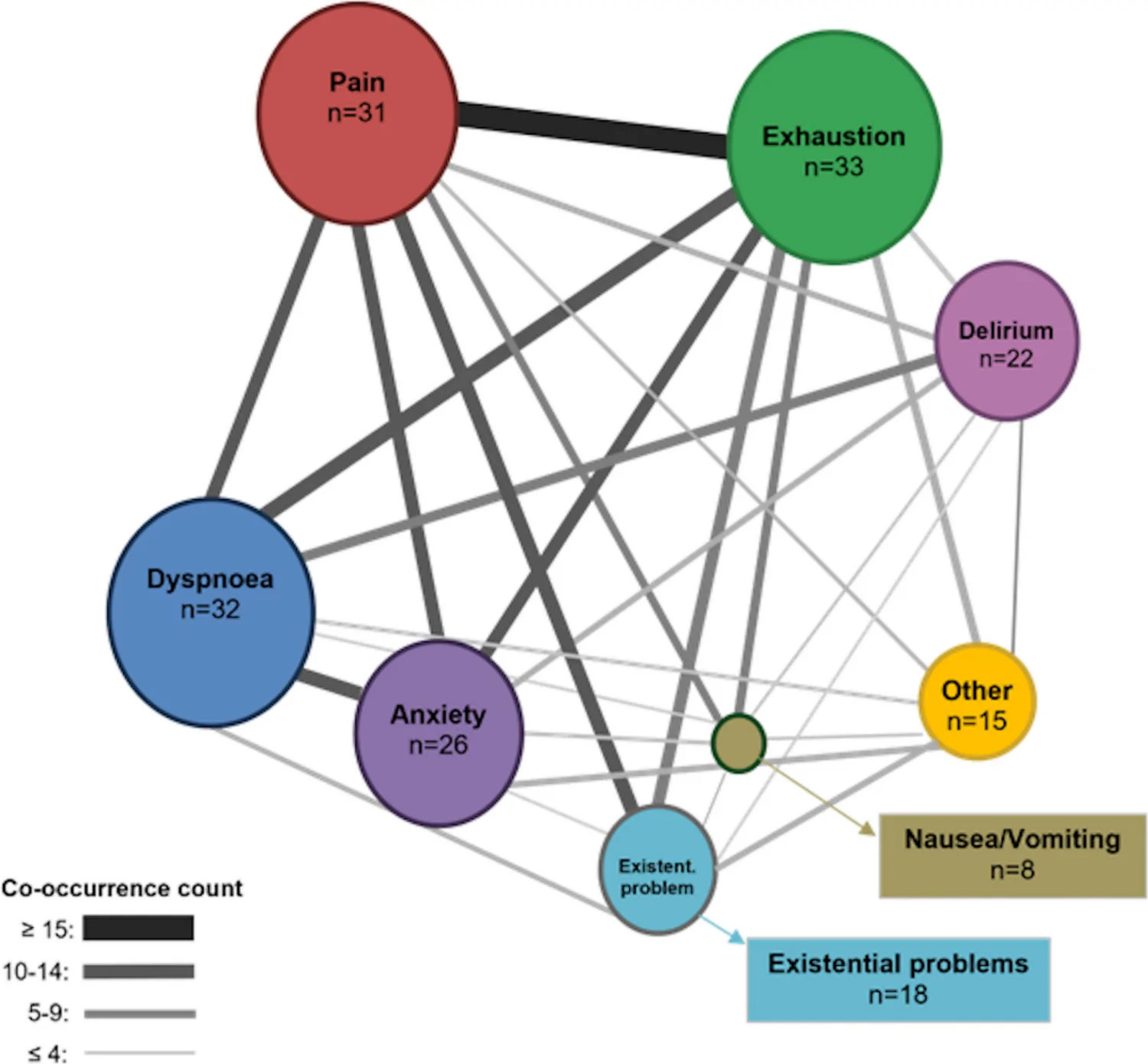
Co-occurrence of refractory symptoms as reported by the HCP (*n = *80). Each node represents the occurrence of symptoms (numbers), and lines between nodes show co-occurrence of symptom pairs; thicker lines indicate more frequent co-occurrence (count); *HCP* health care professional

Only 19% of the patients had a single refractory symptom, 32 (40%) had two and in more than 40% of the sample (*n = *33) ≥ 3 refractory symptoms were reported in various combinations. Exhaustion commonly co-occurred with pain (*n = *19, 24%), dyspnoea (*n = *12, 15%), anxiety (*n = *10, 13%), and existential suffering (*n = *9, 11%; see Fig. 2). Anxiety was commonly reported alongside dyspnoea (*n = *11, 14%), and pain (*n = *10, 13%). Both exhaustion (*n = *33, 41%) and existential suffering (*n = *18, 23%) were never reported as the sole reason for initiating palliative sedation.

Free-text descriptions of exhaustion referred to profound physical and/or mental depletion, often accompanied by an inability to cope with the burden of illness. Descriptions of existential suffering included loss of autonomy, increasing dependency, reduced quality or meaning of life, and fear.

### Patient- or proxy-reported symptom burden (ESAS)

**Fig. 3:**
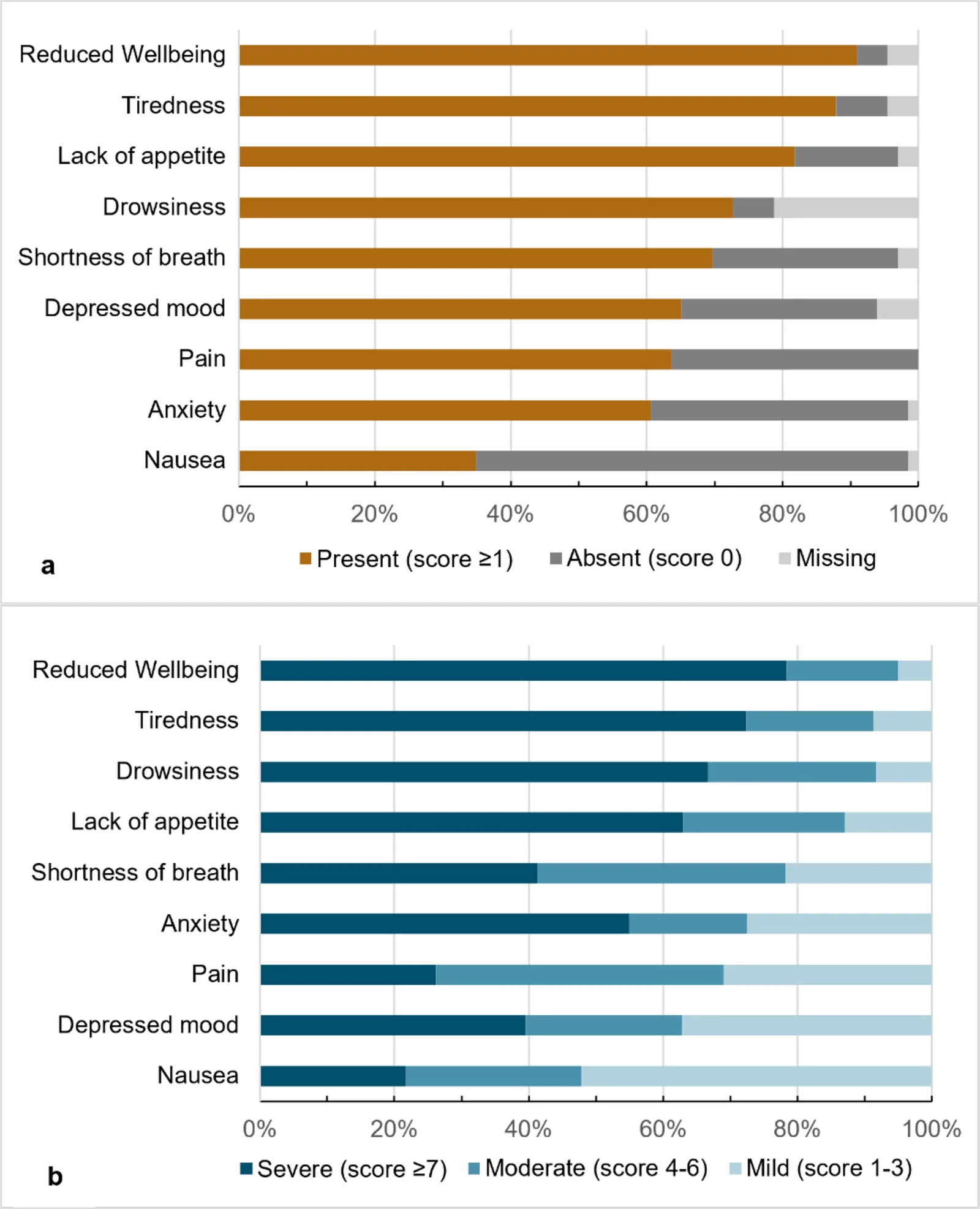
Patient- or proxy-reported symptom burden (ESAS) in the ≤ 3 days before PS. ESAS measurements were completed by 46 patients and 20 proxies. **a** Scored presence (≥ 1) or absence (0) of the nine symptoms at the ESAS. Missing items for: drowsiness, *n = *14, depressed mood, *n = *4; wellbeing and tiredness, *n = *3; breath and appetite, *n = *2; anxiety and nausea, *n = *1. **b** Scored intensity of the present symptoms. In 66 measurements, present ESAS symptoms were: reduced feeling of wellbeing, *n = *60; tiredness, *n = *58; lack of appetite, *n = *46; drowsiness, *n = *39; shortness of breath, *n = *36; depressed mood, *n = *36; pain, *n = *34; anxiety, *n = *30; and nausea, *n = *22. *PS* Palliative Sedation, *ESAS* Edmonton Symptom Assessment System

ESAS assessments were available for 66/80 patients (83%) within three days before initiation of sedation (median 0.9 days, IQR 0.7–1.8). Patients completed 70% (*n = *46) of assessments themselves. In most cases (*n = *48, 73%), all nine ESAS items were completed. In thirteen assessments (20%), eight items were recorded, as the local ESAS did not include drowsiness. In five patients (8%), multiple items were missing (See Fig. 3). All symptoms except nausea were reported to be present in more than 60% of cases (range, 61–91%, Fig. 3a). Reduced sense of wellbeing (91%), tiredness (88%), appetite loss (82%), and drowsiness (73%) were most prevalent and most often rated ≥ 4. As shown in Fig. 3b, severe burden (≥ 7) occurred across all symptoms.

### Sensitivity analysis of patient-reported ESAS assessments

Sensitivity analysis of patient-reported ESAS (*n = *46) showed similar distributions, including reduced wellbeing. In comparison with the total sample (including proxy assessments), a higher proportion of patients rated depressed mood as clinically relevant (≥ 4; 75% vs. 63%), whereas nausea was reported less frequently (42% vs. 48%).

Data are not shown in table.

### Medication use 24 h before palliative sedation

Patients received a median of six medications during the final 24 h of life. Most patients received analgesics, predominantly opioids (91%), whereas antihistamines (11%) and antidepressants (5%) were less commonly used. Patients with fewer symptoms (FSG; *n = *47) more frequently received anti-ulcer agents, corticosteroids, cardiovascular drugs, and anti-infectives compared to patients in the MSG (≥ 3 symptoms, *n = *33). Conversely, the MSG patients were more often treated with anxiolytics, hypnotics, and sedatives. Detailed information on medications administered prior to sedation is presented in the Supplemental Table 3.

### Medication use and other clinical characteristics during palliative sedation

Following the initiation of palliative sedation, clinical practice was largely uniform across patients. Most medications were discontinued, while opioids were predominantly continued or adjusted (93%; Supplemental Table 3). Sedation practices also showed consistency, as detailed in Table 2. Continuous midazolam was the primary used sedative, with similar starting doses between groups (2.0 vs 2.3 mg/hour). Additional or alternative sedatives were administered in 32% versus 48% of patients. The median duration of palliative sedation was comparable in both groups (approximately 36–37 h). No significant differences were observed regarding concomitant medications during palliative sedation or other evaluated parameters.

**Table 2:** PS characteristics compared for patients with fewer or more symptoms

	FSG (1–2 symptoms) *n = 47*	MSG (≥ 3 symptoms) *n = 33*	p value
Time from admission to start PS, *in days*	13.8 [8.1–23.7]	16.6 [8.5–43.8]	0.2^a^
Modality of palliative sedation			0.4^b^
*Continuous*	43 (91%)	32 (97%)	
*Intermittent changed in continuous*	3 (6%)	0	
*Intermittent*	1 (2%)	1 (3%)	
Sedative Medication used for PS			0.2^b^
*Midazolam only*	32 (68%)	17 (52%)	
*Midazolam in combination with other*	15 (32%)	16 (48%)	
Midazolam dosage first 6 h*, in mg/hr*	2.0 [1.0–3.4]	2.3 [1.3–3.6]	0.2^a^
Time to reach adequate sedation*, in minutes*	30 [15–120]	60 [30–120]	1.0^a^
Duration PS, *in hours*	36.5 [15.6–86.6]	37.0 [16.2–68.3]	0.9^a^

Variables are presented in median [IQR] or numbers (%). *PS* palliative sedation, *IQR* Interquartile range, *FSG* Fewer Symptoms Group, *MSG* More Symptoms Group

a. Mann–Whitney U test; b. Fisher's Exact Test

## Discussion

In patients with advanced cancer, symptoms were systematically evaluated by both healthcare professionals and patients prior to palliative sedation. During the final three days before palliative sedation, ESAS assessments showed reduced feeling of wellbeing, fatigue, and loss of appetite to be the most prevalent and severe symptoms. Healthcare professionals most frequently recorded the co-occurrence of multiple symptoms in individual patients. Differences were observed in medication use prior to sedation between patients with fewer versus more symptoms, although these did not extend to differences medication use during and clinical application of palliative sedation itself.

The prevalence and severity of symptoms assessed using the ESAS correspond with findings from previous studies on end-of-life experiences in cancer patients [16, 17]. Moreover, the symptoms most frequently reported by our cohort were also the most intense ones scored on the ESAS, consistent with results reported by de Graaf et al. [18]. Healthcare professionals reported the occurrence of symptoms such as exhaustion, dyspnoea, and pain at the end of life, while refractory dyspnoea and delirium were the most important reasons for initiating palliative sedation, again in line with earlier research [1, 7].

Our findings provide important insights into the underlying nature of refractory suffering. They support the notion that such suffering in the context of end-of-life care often results from the co-occurrence of several symptoms rather than from a single dominant one. While Ferraz Gonçalves et al.[10] proposed that symptom cut-off points could help determine when suffering becomes refractory and palliative sedation might be indicated, our results suggest that such thresholds fail to capture the complex and multifactorial character of suffering at the end of life. In our patients, a self-reported decline in overall wellbeing, often accompanied by profound exhaustion, reflected a state of global, multidimensional suffering rather than an isolated refractory symptom. This overall decline should not be interpreted as a purely subjective trigger for palliative sedation, but rather as a clinical signal prompting careful multidisciplinary evaluation of whether symptom-focused treatments have been exhausted. In this sense, our interpretation aligns with Dong et al. [19], who demonstrated that clusters of emotional and physical symptoms are closely associated with overall quality of life in the terminal phase.

Building on these quantitative findings, healthcare professionals’ free-text descriptions of exhaustion and existential suffering provided additional context on patients’ suffering in the period preceding palliative sedation. In the present study, ‘exhaustion’ was used as an umbrella concept referring to profound physical and mental fatigue. Although terminology may vary across languages and clinical contexts, similar terms and descriptions were observed among healthcare professionals in our sample from different countries and clinical settings. Collectively, these descriptions complement the observed decline in wellbeing and illustrate the subjective and multidimensional nature of refractory suffering. This is consistent with the EAPC framework of palliative sedation, which acknowledges the patient’s experience of intolerable suffering as a central element in clinical decision-making [8]. This multidimensional nature of suffering is also reflected in changes in pharmacological management as patients approach palliative sedation.

Patterns of medication use before sedation offer additional insight into clinical responses to increasing frailty and symptom burden. We hypothesised that patients experiencing multiple concurrent symptoms are generally frailer, prompting discontinuation of non-beneficial medications and a shift towards comfort-focused care [20, 21]. Indeed, in the multiple-symptom group, certain agents such as anti-infectives, corticosteroids, and cardiovascular drugs were used less frequently, in line with palliative care recommendations. Conversely, benzodiazepines for anxiety and sleep were administered more often. Oral agents for which no alternative route was available, such as laxatives, were commonly discontinued because patients were no longer able to take them. These patterns suggest that the presence of multiple symptoms may help clinicians recognise the transition to the dying phase earlier and withdraw futile treatments accordingly [22]. Despite some variation in medication use before, the implementation of palliative sedation itself was consistent across our cohort, in contrast to previous reports describing variability in sedation practices [23]. Opioids were appropriately continued during sedation, in accordance with international guidelines [8]. The uniformity of sedation practices across palliative care settings and countries suggests that sedation depth and medication choices were appropriately tailored to patients’ clinical responses, reflecting adherence to shared clinical principles despite contextual differences. This consistency may be related to the fact that, in our study, palliative sedation was monitored in settings with palliative care experts.

### Strengths and limitations

A key strength of this study lies in the systematic documentation of both clinical and patient-reported symptoms prior to sedation across multiple international palliative care settings. This design provided a nuanced understanding of the transition from active treatment to comfort-focused care. However, several limitations should be acknowledged. First, the absence of a comparison group of patients following other end-of-life trajectories limits the interpretability of our findings. Second, data were lacking on clinicians’ underlying intentions regarding medication prescriptions before palliative sedation, leaving uncertainty as to whether some medications were intended to be discontinued.

Future research should move beyond a narrow biomedical focus and recognise the presence of refractory symptom clusters rather than isolated symptoms. A longitudinal mixed-methods design would be well suited for this purpose, combining quantitative data on patient-reported outcome measures during admission with detailed documentation of pharmacological treatment and goals. Complementary qualitative inquiry should further explore how refractory suffering is interpreted by both patients and clinicians, with particular attention to more subjective states such as exhaustion and existential suffering, including their terminology, conceptual boundaries, and interpretation across different clinical, linguistic, and cultural contexts.

## Conclusion

Refractory suffering in patients with advanced disease is a multidimensional and multifactorial phenomenon, typically arising from interacting symptom clusters rather than a single dominant symptom. A marked decline in wellbeing and increasing symptom burden often reflects this overall state of suffering and may serve as a clinical cue to re-evaluate treatment goals and initiate proactive discussions about possible end-of-life scenarios. A personalised, multidisciplinary approach that integrates physical, psychological, and existential perspectives, and actively involves both patients and relatives, is essential for ethically sound and compassionate care at the end of life of patients with advanced disease.

## Supplementary Information

Below is the link to the electronic supplementary material.

Supplementary file1 (DOCX 22.7 KB)

## Data Availability

Data collected for the study and deidentified for all patients will be made available to other researchers. The study protocol and clinical study report with a summary of study outcomes will also be made available. Data will be available in the Radboud Repository, but without investigator support other than deposited metadata.
