# Supplementary material for: Symptom burden profiles and applied medication strategies in the days before and after initiation of palliative sedation in advanced cancer patients: an international prospective multicentre observational study (PALSED)
Source: Support Care Cancer. 2026 Oct 27;34(11):1160. doi: 10.1007/s00520-026-11358-7 (PMC13714141; doi:10.1007/s00520-026-11358-7)
Supplement: Supplementary file 1 [file 520_2026_11358_MOESM1_ESM.docx]

**Table 3**. Administered medication in 24 hours before and first 24 hours after the start of PS compared for groups with fewer and more refractory symptoms

|  | **Total Sample**  n=80 | | **FSG (1-2 symptoms)**  n=47 | | **MSG (≥3 symptoms)**  n=33 | | ***p value*** | |
| --- | --- | --- | --- | --- | --- | --- | --- | --- |
|  | *Before PS* | *Initiation* | *Before PS* | *Initiation* | *Before PS* | *Initiation* | *Before PS* | *Initiation* |
| Number of medications per patient | 6 [4-8] | 3 [2-4] | 7 [5-9] | 3 [2-4] | 6 [3-7] | 2 [1-4] | **0.03_a_*** | 0.08_a_ |
| Opioids | 73 (91%) | 74 (93%) | 41 (87%) | 43 (91%) | 32 (97%) | 31 (94%) | 0.2_b_ | 1_b_ |
| Other Analgesics | 28 (35%) | 21 (26%) | 17 (36%) | 14 (30%) | 11 (33%) | 7 (21%) | 0.8_c_ | 0.4_c_ |
| Anxiolytics, hypnotics, sedatives | 37 (46%) | NA | 16 (34%) | NA | 21 (64%) | NA | **0.009_c_**** | NA |
| Anti-emetics | 30 (38%) | 26 (33%) | 17 (36%) | 17 (36%) | 13 (39%) | 9 (27%) | 0.8_c_ | 0.4_c_ |
| Corticosteroids, systemic | 38 (48%) | 11 (14%) | 28 (60%) | 9 (19%) | 10 (30%) | 2 (6%) | **0.01_c_*** | 0.1_b_ |
| Anti-psychotics | 37 (46%) | 13 (16%) | 22 (47%) | 8 (17%) | 15 (45%) | 5 (15%) | 0.9_c_ | 0.8_c_ |
| Anti-ulcer agents | 39 (49%) | 9 (11%) | 30 (64%) | 8 (17%) | 9 (27%) | 1 (3%) | **0.001_c_**** | 0.07_b_ |
| Laxatives | 13 (16%) | 0 | 9 (19%) | 0 | 4 (12%) | 0 | 0.5_b_ | NA |
| Antihistamines | 9 (11%) | 1 (1%) | 7 (15%) | 0 | 2 (6%) | 1 (3%) | 0.3_b_ | 0.4_c_ |
| Anti-depressants | 4 (5%) | 0 | 3 (6%) | 0 | 1 (3%) | 0 | 0.6_b_ | NA |
| Cardiovascular treatment | 33 (41%) | 12 (15%) | 26 (55%) | 10 (21%) | 7 (21%) | 2 (6%) | **0.002_c_**** | 0.1_b_ |
| Antithrombotic agents | 26 (33%) | 4 (5%) | 18 (38%) | 2 (4%) | 8 (24%) | 2 (6%) | 0.2_c_ | 1_b_ |
| Anti-infectives | 16 (20%) | 2 (3%) | 13 (28%) | 1 (2%) | 3 (9%) | 1 (3%) | **0.04_c_*** | 1_c_ |
| Antiepileptics | 6 (8%) | 3 (4%) | 3 (6%) | 1 (2%) | 3 (9%) | 2 (6%) | 0.7_b_ | 0.6_b_ |
| Supplements | 11 (14%) | 0 | 8 (17%) | 0 | 3 (9%) | 0 | 0.5_b_ | NA |
| Other medication | 39 (49%) | 27 (34%) | 24 (51%) | 18 (38%) | 15 (45%) | 9 (27%) | 0.6_c_ | 0.3_c_ |

PS: Palliative Sedation; FSG: Fewer Symptoms Group; MSG: More Symptoms Group; IQR: Interquartile range.

Columns: Before PS= last 24 hours before PS was started; Initiation= first 24 hours after PS was started.

a. Mann–Whitney U test; b. Fisher's Exact Test; c. Pearson's Chi-squared test. Significance: *<0.05, **<0.01.
